# 

**Published:** 1893-09-02

**Authors:** 


**OUR NEW OFFICES,**

428, Strand, W.C., will henceforth be the Office of this Journal.

**Notices of Births, Deaths, and Marriages** are inserted in this column at a charge of 2s. 6d. for an announcement not exceeding four lines.

### NOTICE TO CORRESPONDENTS.

All MS., Letters, Books for Review, and other matters intended for the Editor should be addressed THE EDITOR, THE LODGE, PORCHESTER SQUARE, LONDON, W.

The Editor cannot undertake to return rejected MS., even when accompanied by stamped directed envelope.

**Notice to Advertisers and Subscribers.**

All Advertisements, Orders for Copies of THE HOSPITAL, and Business Communications should be addressed to The Publisher, *not to the Editor*, THE HOSPITAL, 428, Strand, W.C.

The Cost of Subscription, post free, is as follows:—Per week, 2½d.; per quarter, 2s. 6d.; per half-year, 5s.; per annum, 8s. 6d. Foreign:—Per Annum, 10s. 8d.; payable, in all cases, in advance.

**"Burdett's Hospital Annual," 1893.**

Fourth year of publication. 700 pp. Boards, gilt lettering. A most complete guide to British, Colonial, Indian, and American Hospitals, Dispensaries, Nursing and Convalescent Institutions, and Asylums. Price 5s. Published by THE SCIENTIFIC PRESS (Limited), 428, Strand, W.C.

**NOTICE.—The Index for Vol. XIII. (ending March, 1893) is on sale at the Office of this Journal, price 2½d., post free.**

### Scraps and Gleanings.

The treasurer's account of the Royal Cornwall Infirmary shows the expenditure to be a little in excess of the ordinary income.

By the will of the late Edwin Booth, 5,000 dollars have been bequeathed to the Home for Incurables at West Farms, U.S.A.

During a short stay at Southport Mr. Arthur Roberts has turned his time to good account, having raised £26 in collections towards the funds of the establishment.

It has been considered desirable to establish a joint hospital for infectious diseases for the districts of Malvern, and Malvern Link urban sanitary authorities.

The Balfour Hospital at Orkney treated sixty-one patients from surrounding parishes during the past year, the revenue of the institution for which period amounts to £455.

The financial condition of the Barton Cottage Hospital continues satisfactory, the balance-sheet on the treasurer's as well as on the house accounts giving a favourable report.

The Lloyd Cottage Hospital at Bridlington (Yorks) presented an exceptionally satisfactory report at the recent annual meeting, the accounts showing a balance of £908 to the good.

Within the last ten years the number of patients has nearly doubled in the Essex and Colchester Hospital; and additional accommodation is, therefore, proposed to meet the requirements of the situation.

The annual Hospital Sunday in connection with the friendly societies of Bromsgrove and neighbourhood took place in the first week of August. This collection added £17 to the funds of the Cottage Hospital.

Very energetic efforts were made by the townsfolk of Dover to raise money for their hospital. On Hospital Sunday the friendly societies and lifeboat men with four bands paraded the town gathering subscriptions.

Miss Florence Nightingale has just celebrated her 73rd birthday, and although ill-health has confined her to her room for many years, yet she is still ceaselessly at work about the welfare of her fellow creatures.

One outcome of Bishop Blyth's mission is the suggestion to build an English hospital at Haifa (Palesetine). The services of the staff have already been offered gratuitously; but for the cost of the building, £2,000 is yet needed.

Lord Penryn, as a contribution towards the funds of the new Infectious Diseases Hospital at Bangor, has returned the purchase-money of the site which has been chosen for the building of the institution, on his estate.

A church parade of the friendly societies was held recently at Seaford, in accordance with an ancient custom in connection with Hospital Sunday. Collections amounting to £8 were given towards the funds of the Sussex County Hospital.

The total amount collected at Warwick on Hospital Saturday this year reached a higher figure than has been the case since 1890. The balance, after paying expenses, was divided between the Warneforth Hospital and the Warwick Cottage Hospital.

A house-to-house collection was made early in August on behalf of the Savernake Cottage Hospital and the Royal Berkshire Hospital, and on the same evening a fire brigade demonstration was arranged for, as also an illuminated cycle parade.

### Medical Vacancies and Appointments.

**APPOINTMENTS.**

J. L. Adam, M.B., C.M.Aberd., reappointed Honorary Surgeon to the Lewes Victoria Hospital. H. S. Ballance, M.B., B.S.Lond., M.R.C.S.Eng., appointed Resident Medical Officer to the Hospital for Consumption and Diseases of the Chest, Brompton. H. P. D'A. Benson, M.B.Edin., appointed Medical Officer for the Kentchurch District and the Workhouse of the Dore Union. W. B. Betenson, L.R.C.P.Lond., M.R.C.S.Eng., appointed Medical Officer for the Second District of the Deepwade Union. E. J. W. Carruthers, M.B., C.M.Edin., appointed Junior Resident Medical Officer to the Hospital for Sick Children, Pendlebury, Manchester. H. Corbett, M.D., appointed Medical Officer for the Kliloughey Dispensary District. G. W. Drabble, appointed Medical Officer of the Walton District of the Chertsey Union. A. T. Drake, M.B., L.R.C.S.I., appointed Medical Officer for the Fourth District of the Greenwich Union. E. C. Edwards, M.B., C.M.Edin., appointed Assistant House Surgeon to the East Suffolk Hospital, Ipswich. W. H. Fawcett, L.R.C.P., L.R.C.S.Edin., appointed Medical Officer for the Second District and Workhouse of the Wimborne and Cranborne Union. C. B. Hillyar, appointed Medical Officer for the Milton Abbott District of the Tavistock Union. A. Jamison, M.A., M.B., B.Ch., B.A.O., appointed House Surgeon to the Bristol Hospital for Sick Children and Women. D. O. Kerr, M.B., C.M.Edin., reappointed Medical Officer for the Keelby District of the Grimsby Union. W. H. Maling, M.R.C.S., L.S.A., appointed Honorary Surgeon to the Sunderland and North Durham Eye Infirmary. M. O. Maurice, L.R.C.P.Lond., M.R.C.S., appointed Resident Clinical Assistant to the St. Marylebone Infirmary, Notting Hill. P. Mitchell, M.D.Aberd., reappointed Parochial Medical Officer for Oyne. C. E. Oldacres, M.R.C.S.Eng., L.R.C.P.Edin., appointed Medical Officer of the Workhouse of the Daventry Union. H. E. Owen, L.R.C.P.Lond., appointed Medical Officer of Health for the Kingsbridge Urban Sanitary District. W. E. Price, M.D.Durh., M.R.C.S.Eng., appointed Medical Officer for the Workhouse of the Ross Union. G. E. Rennie, B.A.Syd., M.D.Lond., M.R.C.S.Eng., appointed Government Pathologist, Sydney, New South Wales. M. A. Robinson, L.R.C.P., L.R.C.S.Edin., appointed Medical Officer for the Seventh District of the Deepwade Union. B. M. H. Rogers, M.B., B.Ch., M.R.C.S., L.R.C.P., appointed Physician to the Bristol Hospital for Sick Children and Women. G. Rowell, F.R.C.S., appointed Assistant Anæsthetist to King's College Hospital. G. H. Snowden, M.R.C.S.Eng., appointed Medical Officer for the Halford District of the Shipston-on-Stour Union. J. W. Stephens, L.R.C.P.Lond., M.R.C.S.Eng., appointed Medical Officer of Health for the Cardigan Urban and Port Sanitary Authority, and Certifying Surgeon under the Factory Acts, and Inspector of Seamen for Cardigan. A. E. Thorpe, L.R.C.P., L.R.C.S.Edin., appointed Medical Officer for the Second District of the Newport (Salop) Union. W. O. Trotter, L.R.C.P.Edin., M.R.C.S.Eng., appointed Medical Officer for the Brandon District of the Thetford Union. J. S. Vassalli, L.R.C.P., L.R.C.S.Edin., L.F.P.S.Glasg., appointed Medical Officer and Public Vaccinator for the Western Sanitary District of the Scarborough Union. T. Webster, M.R.C.S.Eng., L.S.A., appointed Medical Officer for the Bewdley District of the Kidderminster Union. G. G. D. Willet, M.R.C.S.Eng., reappointed Medical Officer for the Bilton and Merksbury Sanitary District of the Keynsham Union. C. Wintle, M.R.C.S., L.R.C.P., appointed Surgical Registrar and Out-Patients' Assistant to the Bristol Hospital for Sick Children and Women.

### VACANCIES.

The following vacancies are announced this week, the amount of salary in each case being given after the name of the institution:

### Resident Medical Officers.

Brighton, Hove, and Preston Dispensary, Board Room, Queen's Road, Brighton. House Surgeon, unmarried, doubly qualified. Salary £140 per annum, with furnished apartments, coal, gas, and attendance. Applications to C. Somers Clarke, Honorary Secretary, by September 1st.

General Hospital, Birmingham. House Physician. Salary £70 per annum, with residence, board, and washing. Applications to Howard J. Collins, House Governor, by September 2nd.

City of London Hospital for Diseases of the Chest, Victoria Park, E. House Physician. Appointment for six months. Board, residence, and allowance for washing provided. Applications to the Secretary, at the Office, 24, Finsbury Circus, E.C., by September 24th.

Kent County Asylum, Chartham, near Canterbury. Junior Assistant Medical Officer, unmarried. Salary £125 per annum, with furnished apartments, board, and attendance. Applications to the Clerk of the Committee of Visitors, Allen Fielding, Solicitor, Canterbury, by September 20th

St. Peter's Hospital for Stone and Urinary Diseases, Henrietta Street, Covent Garden. House Surgeon; must be M.R.C.S.; appointment for six months. Honorarium 25 guineas, with board, lodging, and washing. Applications to the Secretary by September 11th.

Stafford General Infirmary, Stafford. Assistant House Surgeon. Board and residence provided. Applications to the Secretary by September 9th.

### Non-Resident Medical Officers.

Borough of Chesterfield. Medical Officer of Health for the Urban Sanitary District. Salary £200 per annum. Applications endorsed "Medical Officer" to John Middleton, Town Clerk, by September 4th.

University of Aberdeen. Eleven Examiners in Medicine. The Examiners in Medicine and Surgery will receive a grant of £50 each, and the other nine a grant of £40 each. Applications to Robert Walker, Secretary, the University Court, by September 25th.

### PASS LIST.

### Royal College of Surgeons of Edinburgh.

The following gentlemen, having passed the requisite examination, have been elected fellows of the college: J. Fuller, L.R.C.S.Eng., M.R.C.P.Irel., Long Ashton, Bristol; E. B. Fuller, M.B., C.M.Edin., L.R.C.S.Eng., Cape Town; R. J. Pope, M.D., L.R.C.S.Eng., 19, Coates Crescent, Edinburgh; E. R. Morton, L.R.C.P. and S.Eng., &c., City Hospital, Edinburgh; J. C. Gibb Macnab, M.B., C.M., L.R.C.S.Eng., Dysart; J. A. Lea, L.R.C.P.Edin. and Lond., M.R.C.S.Eng., Carnarvon Hospital, Kimberley, Cape Colony; A. C. Hartley, M.D., L.R.C.S.Eng., 12, Victoria Terrace, Bedford; and J. Hardie, M.B., C.M., L.R.C.S.Eng., 12, Newington Road, Edinburgh.

### EXAMINATION IN EDINBURGH FOR THE TRIPLE QUALIFICATION.

### PAPERS SET IN THE SECOND EXAMINATION

Questions in Physiology (all of which are to be answered).

1. What are the various functions of the Pneumogastric Nerve?
2. What changes are produced in Food by (a) the Gastric Juice, (b) the Pancreatic Juice?
3. What action has the Endothelium of the Blood Vessels on the Blood? Describe fully what changes occur in the Blood when it is removed from the body.
4. What is the specific gravity of Urine? Mention any circumstances which cause the specific gravity to vary. How much urea is excreted daily?

Questions in Materia Medica (all of which are to be answered).

1. Describe the official Hypodermic injections.
2. Enumerate the official preparations of Rhubarb, and state their doses.
3. Mention the characters of Antimonium Tartaratum, Sodii, Biboras and Amyl Nitris, stating also in what doses they are administered.

